# A Neuro-Fuzzy Approach in the Classification of Students' Academic Performance

**DOI:** 10.1155/2013/179097

**Published:** 2013-11-04

**Authors:** Quang Hung Do, Jeng-Fung Chen

**Affiliations:** Department of Industrial Engineering and Systems Management, Feng Chia University, No. 100, Wenhwa Road, Taichung 40724, Taiwan

## Abstract

Classifying the student academic performance with high accuracy facilitates admission decisions and enhances educational services at educational institutions. The purpose of this paper is to present a neuro-fuzzy approach for classifying students into different groups. The neuro-fuzzy classifier used previous exam results and other related factors as input variables and labeled students based on their expected academic performance. The results showed that the proposed approach achieved a high accuracy. The results were also compared with those obtained from other well-known classification approaches, including support vector machine, Naive Bayes, neural network, and decision tree approaches. The comparative analysis indicated that the neuro-fuzzy approach performed better than the others. It is expected that this work may be used to support student admission procedures and to strengthen the services of educational institutions.

## 1. Introduction

Accurately predicting student performance is useful in many different contexts in educational environments. When admission officers review applications, accurate predictions help them to distinguish between suitable and unsuitable candidates for an academic program. The failure to perform an accurate admission decision may result in an unsuitable candidate being admitted to the university. Since the quality of an educational institution is mainly reflected in its research and training, the quality of admitted candidates affects the quality level of an institution. Accurate prediction enables educational managers to improve student academic performance by offering students additional support such as customized assistance and tutoring resources. The results of prediction can also be used by lecturers to specify the most suitable teaching actions for each group of students and provide them with further assistance tailored to their needs. Thus, accurate prediction of student achievement is one way to enhance quality and provide better educational services. As a result, the ability to predict students' academic performance is important for educational institutions. A very promising tool to achieve this objective is the use of data mining. Data mining processes large amounts of data to discover hidden patterns and relationships that support decision-making.

Data mining in higher education is forming a new research field called educational data mining [1, 2]. The application of data mining to education allows educators to discover new and useful knowledge about students [3]. Educational data mining develops techniques for exploring the types of data that come from educational institutions. There are several data mining techniques, such as statistics and visualization, clustering, classification, and outlier detection. Among these, classification is one of the most frequently studied techniques. Classification is a process of supervised learning where data is separated into different classes. Classification maps data into predefined groups of classes. The goal of a classification model is to predict the target class for each sample in the dataset. There are various approaches for classification of data, including support vector machine (SVM), artificial neural network (ANN), and Bayesian classifier approaches [4]. Based on these approaches, a classification model that describes and distinguishes data classes is constructed. Then, the developed model is used to predict the class label of new data that does not belong to the training dataset. These approaches have been widely applied in educational environments [5–7]. In this study, we present a classification model based on a neuro-fuzzy approach to predict students' academic performance level.

Neural networks and fuzzy set theory, which are termed soft computing techniques, are tools of establishing intelligent systems. A fuzzy inference system (FIS) employing fuzzy if-then rules in acquiring knowledge from human experts can deal with imprecise and vague problems [8]. FISs have been widely used in many applications including optimization, control, and system identification. Fuzzy systems do not usually learn and adjust themselves [9], whereas a neural network (NN) has the capacity to learn from its environment, self-organize, and adapt in an interactive way. For these reasons, a neuro-fuzzy system, which is the combination of fuzzy inference system and neural network, has been introduced to produce a complete fuzzy-rule-based system [10, 11]. The merits of neural networks and fuzzy systems can be integrated in a neuro-fuzzy approach. Fundamentally, a neuro-fuzzy system is a fuzzy network that not only includes a fuzzy inference system but can also overcome some limitations of neural networks, as well as the limits of fuzzy systems [12, 13] because it can learn and represent knowledge in an interpretable manner and learning ability. One of the neuro-fuzzy systems, a neuro-fuzzy classifier (NFC), combines the powerful description of FIS with the learning capabilities of NNs to partition a feature space into classes. NFCs have been commonly used for different problems [14, 15]. In this paper, we use an NFC with a scaled conjugate gradient (SCG) algorithm improved by Cetişli and Barkana [16] to classify students based on their expected academic performance levels.

The paper is organized into seven sections. After the introduction in Section 1, some of the most commonly classification approaches are presented in Section 2.  Section 3 describes the neuro-fuzzy classifier.  Section 4 is dedicated to describing the process of NFC training. The data preparation is in Section 5 and the results are in Section 6. Finally, Section 7 presents the conclusion.

## 2. Classification Approaches

Various approaches are used for discovering knowledge from databases. In this section, the most commonly used approaches are briefly discussed.

### 2.1. Support Vector Machine (SVM)

SVM is a supervised learning method influenced by advances in statistical learning theory [17]. SVM has been successfully applied to number of applications in classification and recognition problems. Using training data, SVM maps the input space into a high-dimensional feature space. In the feature space, the optimal hyper-plane is identified by maximizing the margins or distances of class boundaries. The training points that are closest to the optimal hyper-plane are called support vectors. When the decision surface is achieved, it can be used for classifying new data.

Consider a training dataset of feature-label pairs (*x*_*i*_, *y*_*i*_) with *i* = 1,…, *n*. The optimum separating hyper-plane is represented as

(1) $$\begin{array}{c} g\left(x\right)=\mathrm{sign}\left(\overset{n}{\underset{i=1}{\sum }}y_{i}\alpha_{i}K\left(x_{i},x_{j}\right)+b\right), \end{array}$$

where *K*(*x*_*i*_, *x*_*j*_) is the kernel function; *α*_*i*_ is a Lagrange multiplier; and *b* is the offset of the hyper-plane from the origin. This is subject to constraints 0 ≤ *α*_*i*_ ≤ *C* and ∑_*α*_*i*_*y*_*i*__ = 0, where *α*_*i*_ is a Lagrange multiplier for each training point and *C* is the penalty. Only those training points lying close to the support vectors have nonzero *α*_*i*_. However, in real-world problems, data are noisy and there will be no linear separation in the feature space. Hence, the optimum hyper-plane can be identified as

(2) $$\begin{array}{c} y_{i}\left(w\cdot x_{i}+b\right)\geq 1-\xi_{i},\;\xi_{i}\geq 0, \end{array}$$

where *w* is the weight vector that determines the orientation of the hyper-plane in the feature space and *ξ*_*i*_ is the *i*th positive slack variable that measures the amount of violation from the constraints.

### 2.2. Naive Bayes Classifier

A Naive Bayes classifier is based on Bayes' theorem and the probability that a given data point belongs to a particular class [18]. Assume that we have *m* training samples (*x*_*i*_, *y*_*i*_), where *x*_*i*_ = (*x*_*i*1_, *x*_*i*2_,…, *x*_*in*_) is an *n*-dimensional vector and *y*_*i*_ is the corresponding class. For a new sample *x*_tst_, we wish to predict its class *y*_tst_ using Bayes' theorem:

(3) $$\begin{array}{c} y_{\text{tst}}=\arg\underset{y}{\max}\;P\left(y\;|\;x_{\text{tst}}\right)=\arg\underset{y}{\max}\frac{P\left(x_{\text{tst}}y\right)P\left(y\right)}{P\left(x_{\text{tst}}\right)}. \end{array}$$

However, the above equation requires estimation of distribution *P*(*x* | *y*), which is impossible in some cases. A Naive Bayes classifier makes a strong independence assumption on this probability distribution by the following equation:

(4) $$\begin{array}{c} P\left(x\;|\;y\right)=\overset{n}{\underset{j=1}{\prod }}P\left(x_{j}\;|\;y\right). \end{array}$$

This means that individual components of *x* are conditionally independent given its label *y*. The task of classification now proceeds by estimating *n* one-dimensional distributions *P*(*x*_*j*_ | *y*).

### 2.3. Neural Network (NN)

Neural networks can represent complex relationships between inputs and outputs [19]. The classification procedure based on NNs consists of three steps, namely, data pre-processing, training, and testing. The data pre-processing refers to the feature selection. For the data training, the features from the data preprocessing step are fed to the NN, and a classifier is generated through the NN. Finally, the testing data is used to verify the efficiency of the classifier.

### 2.4. Decision Tree (DT)

A decision tree is a hierarchical model composed of decision rules that recursively split independent inputs into homogenous sections [20]. The aim of constructing a DT is to find the set of decision rules that can be utilized to predict outcomes from a set of input variables. A DT is called a regression or classification tree if the target variables are continuous or discrete, respectively [21]. The computational complexity of a decision tree may be high, but it can help to identify the most important input variables in a dataset by placing them at the top of the tree.

## 3. Neuro-fuzzy Classifier (NFC) Architecture

A typical fuzzy classification rule *R*_*i*_, which demonstrates the relation between the input feature space and classes, is as follows:

 
*R*_*i*_ : if *x*_*s*1_ is *A*_*i*1_ and ⋯*x*_*sj*_ is *A*_*ij*_⋯ and *x*_*sn*_ is *A*_*in*_, then class is *C*_*k*_,

where *x*_*sj*_ represents the *j*th feature or input variable of the *s*th sample; *A*_*ij*_ denotes the fuzzy set of the *j*th feature in the *i*th rule; and *C*_*k*_ represents the *k*th label of class. *A*_*ij*_ is identified by the appropriate membership function [22].

**Figure 1:**
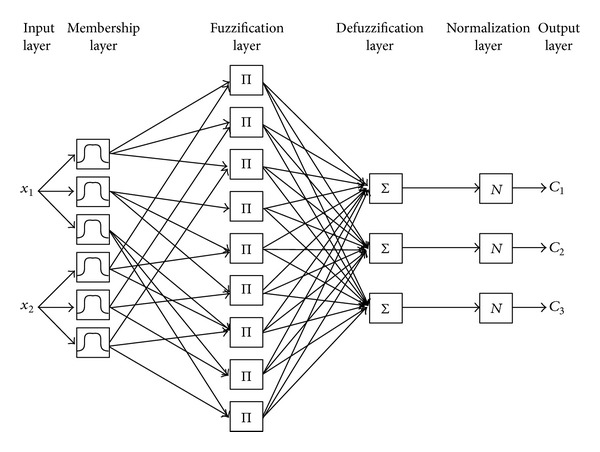
A neuro-fuzzy classifier.

In the NFC, the feature space is partitioned into multiple fuzzy subspaces by fuzzy if-then rules. These fuzzy rules can be represented by a network structure. An NFC is a multilayer feed-forward network consisting of the following layers: input, fuzzy membership, fuzzification, defuzzification, normalization, and output. The classifier has multiple inputs and multiple outputs.  Figure 1 depicts an NFC with two features {*x*_1_, *x*_2_} and three classes {*C*_1_, *C*_2_, *C*_3_}. Every input is defined with three linguistic variables; thus, there are nine fuzzy rules.

Membership layer: the membership function of each input is identified in this layer. Several types of membership functions can be used. In this study, a Gaussian function is utilized, since this function has fewer parameters and smoother partial derivatives for parameters. The Gaussian membership function is defined as

(5) $$\begin{array}{c} \mu_{ij}\left(x_{sj}\right)=\exp\left(-\frac{{\left(x_{sj}-c_{ij}\right)}^{2}}{2\sigma_{ij}^{2}}\right), \end{array}$$

where *μ*_*ij*_(*x*_*sj*_) is the membership grade of *i*th rule and *j*th feature; *x*_*sj*_ represents the *s*th sample and *j*th feature; *c*_*ij*_ and *σ*_*ij*_ are the center and the width of Gaussian function, respectively.

Fuzzification layer: each node in this layer generates a signal corresponding to the degree of fulfillment of the fuzzy rule for the *x*_*s*_ sample. It is called the firing strength of a fuzzy rule with respect to an object to be classified. The firing strength of the *i*th rule is as follows:

(6) $$\begin{array}{c} \alpha_{is}=\overset{N}{\underset{j=1}{\prod }}\mu_{ij}\left(x_{sj}\right), \end{array}$$

where *N* is the number of features.

Defuzzification layer: in this layer, weighted outputs are calculated; each rule affects each class according to their weights. If a rule controls a specific class region, the weight between that rule output and the specific class will be larger than the other weights. Otherwise, the class weights are small. The weighted output for the *s*th sample that belongs to the *k*th class is calculated as follows:

(7) $$\begin{array}{c} \beta_{sk}=\overset{M}{\underset{i=1}{\sum }}\alpha_{is}w_{ik}, \end{array}$$

where *w*_*ik*_ denotes the degree of belonging to the *k*th class that is controlled by the *i*th rule and *M* represents the number of rules.

Normalization layer: the outputs of the network should be normalized, since the summation of weights may be larger than 1 in some cases

(8) $$\begin{array}{c} o_{sk}=\frac{\beta_{sk}}{\sum_{l=1}^{K}\beta_{sl}}, \end{array}$$

where *o*_*sk*_ denotes the normalized value of the *s*th sample that belongs to the *k*th class and *K* is the number of classes.

Then, the class label for the *s*th sample is obtained by the maximum *o*_*sk*_ value as follows:

(9) $$\begin{array}{c} C_{s}=\underset{k=1,2,\ldots ,K}{\max}\left\{o_{sk}\right\}, \end{array}$$

where *C*_*s*_ denotes the class label of the *s*th sample.

## 4. Training NFC

**Figure 2:**
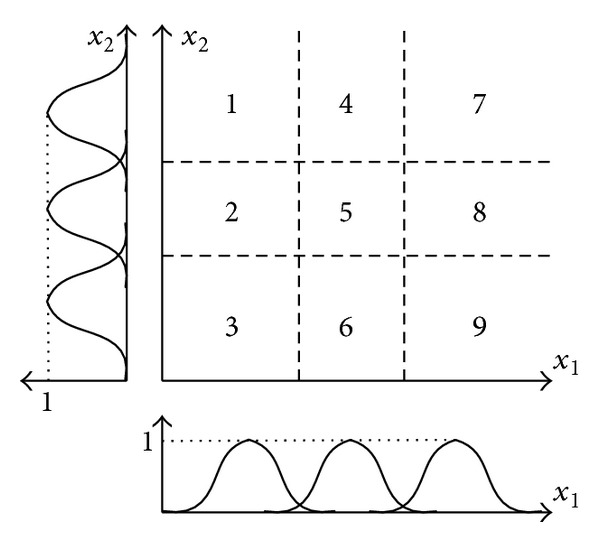
Partition of a feature space with two inputs and three membership functions for each input.

In order to determine an optimum fuzzy region, the parameters, *θ* = {*S*_*M*×*N*_, *C*_*M*×*N*_, *W*_*M*×*K*_}, of the fuzzy if-then rules must be optimized [23], where *S* and *C* are the matrices containing the sigma and centre values, respectively; *W* presents the weight matrix of connections from fuzzification layer to defuzzification layer; *M*, *N*, and *K* are the number of rules, features, and classes, respectively. The *K*-means clustering method is utilized to obtain the initial parameters and to form the fuzzy if-then rules [24]. The *K*-means clustering method aims to partition the input feature space into a number of clusters in which each data point belongs to the cluster with the nearest mean. This results in a partitioning of the data space. For a given dataset, this method can estimate the number of clusters and the cluster centers. In Figure 2, a feature space with two inputs {*x*_1_, *x*_2_} is shown. Suppose that every input is divided into three fuzzy sets by employing the *K*-means method. Each fuzzy set is characterized by the appropriate membership function; as a result, each input has three membership functions. A fuzzy classification rule describes the relationship between the input feature space and the classes. The formation of the fuzzy if-then rules is illustrated in Figure 2. Each input is represented as three membership functions; thus, we have nine fuzzy rules.

Several training algorithms, including the Kalman filter [25], the Levenberg-Marquardt method [26], have been used to optimize the parameters of NFC. Application of the SCG algorithm showed that the SCG algorithm produced the least error and the highest efficiency [27]. Moreover, the SCG improved by Cetişli and Barkana [16] has the ability to decrease the training time per iteration and to not affect the convergence rate. Hence, the improved SCG method is utilized for optimization in this study.

The cost function is determined from the least mean squares of the difference between target value and calculated class value. The cost function *E* is as follows:

(10) $$\begin{array}{c} E=\frac{1}{N}\overset{S}{\underset{s=1}{\sum }}E_{s},\;\;E_{s}=\frac{1}{2}\overset{K}{\underset{k=1}{\sum }}\left(t_{sk}-o_{sk}\right), \end{array}$$

where *S* is the number of samples and *t*_*sk*_ and *o*_*sk*_ represent the target and calculated values of the *s*th sample belonging to the *k*th class, respectively. If the *s*th sample belongs to the *k*th class, the target value *t*_*sk*_ is 1; otherwise, it is 0.

The aim of SCG algorithm is to find the optimal or near-optimal parameter θ* from the cost function *E*(*θ*). In the SCG algorithm, the next closest update vector, *θ*_*t*+1_, to the current vector *θ*_*t*_ is identified as

(11) $$\begin{array}{c} \theta_{t+1}=\theta_{t}-g_{t}H_{t}^{-1}, \end{array}$$

where *g*_*t*_ = *E*′(*θ*_*t*_) and *H*_*t*_ = *E*′′(*θ*_*t*_) are the gradient vector and the Hessian matrix of *E*(*θ*_*t*_), respectively. The product, −*g*_*t*_ *H*_*t*_^−1^, is called the Newton step; its Newton direction is indicated by the minus sign. If the Hessian matrix is positive definite and *E*(*θ*_*t*+1_) is quadratic, Newton's method directly reaches a local minimum in a single step [23]; however, reaching a local minimum commonly requires more iterations. Møller [28] introduced a temporal parameter vector *θ*_*m*,*t*_ which is between *θ*_*t*+1_ and *θ*_*t*_ and is defined as

(12) $$\begin{array}{c} \theta_{m,t}=\theta_{t}+\gamma_{t}d_{t},\;0<\gamma_{t}\ll 1, \end{array}$$

where *γ*_*t*_ is the short step size and *d*_*t*_ = −*g*_*t*_ is the conjugate direction vector of the temporal parameter vector at the *t*th iteration. The actual parameter update is calculated as

(13) $$\begin{array}{c} \theta_{t+1}=\theta_{t}+\alpha_{t}d_{t}, \end{array}$$

where *θ*_*t*+1_ is next parameter update vector; *θ*_*t*_ is current parameter vector; and *α*_*t*_ is actual parameter updating step size and is calculated as follows:

(14) $$\begin{array}{c} \alpha_{t}=\frac{-d_{t}^{T}E^{\prime }\left(\theta_{t}\right)}{d_{t}^{T}s_{t}},\;\;s_{t}=E^{\prime \prime }\left(\theta_{t}\right)d_{t}\approx \frac{E^{\prime }\left(\theta_{m,t}\right)-E^{\prime }\left(\theta_{t}\right)}{\gamma_{t}}, \end{array}$$

where *s*_*t*_ is the second-order information and *α*_*t*_ denotes the basic long step size. To calculate *α*_*t*_, the second-order information *s*_*t*_ should be obtained from the first-order gradients.

In the SCG algorithm, two different gradients of the parameter vector are calculated in any iteration. The gradient *d*_*t*_ of the temporal parameter vector *θ*_*m*,*t*_ is calculated using the short step size *γ*_*t*_, and the gradient of the actual parameter update *θ*_*t*+1_ is calculated using the long-step size *α*_*t*_, which is obtained using *θ*_*m*,*t*_. However, Cetişli and Barkana [16] stated that the gradient of *θ*_*m*,*t*_ in the *t*th iteration is more suitable than the gradient of *θ*_*t*+1_. In the SSCG (speeding up SCG), the second gradient is used to estimate the first gradient for the next iteration. The estimation of only one gradient has the benefit of shortening the iteration time.

## 5. Data Preparation

This section represents application of the proposed model in the prediction of students' academic performance level. In this paper, an application related to the context of Vietnam was used as an illustration.

### 5.1. Identifying Input and Output Variables

Through a literature review and discussion with admission officers and experts, a number of academic, social-economic, and other related factors that are considered to have influence on the students' academic performance were determined and chosen as input variables. The input variables were obtained from the admission registration profile and are as follows: the university entrance exam results (normally, in Vietnam, candidates take three exams for the fixed group of subjects they choose), the overall average score from a high school graduation examination, the elapsed time between graduating from high school and obtaining university admission, the location of high school (there are four regions, as defined by the government of Vietnam: Region 1, Region 2, Region 3, and Region 4. Region 1 includes localities with difficult economic and social conditions; Region 2 includes rural areas; Region 3 includes provincial cities; and Region 4 includes central cities), type of high school attended (private or public), and gender (male or female). Nonnumerical factors must be converted into a format suitable for neural networks. The input variables and ranges are presented in Table 1.

The preliminary step of all classification approaches is to identify the number of classes in which dataset is to be classified and to assign class labels. Based on the current grading system used by the university and the scope of this project, three classes were identified as “good,” “average,” and “poor.” As shown in Table 2, class labels were defined as 1, 2, and 3 for “good,” “average,” and “poor,” respectively.

### 5.2. Dataset

We obtained our data from the University of Transport Technology, which is a public university in Vietnam. For input variables, we used a real dataset from students in the Department of Bridge Construction, and for output variables, we used their achievements for the 2011-2012 academic year. The dataset belongs to the University of Transport Technology and can be requested by contacting the corresponding author by email. The dataset consisted of 653 cases and was divided into two groups. The first group (about 60%) was used for training the model. The second group (about 40%) was employed for testing the model. The training dataset served in model building while the other group was used for the validation of the developed model.

## 6. Results

**Figure 3:**
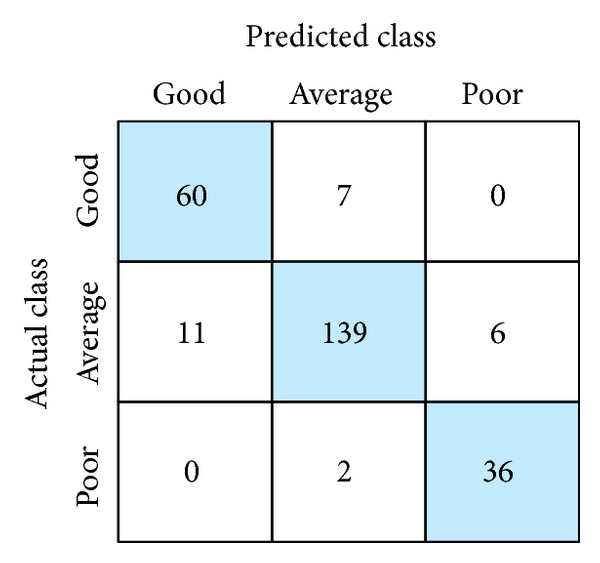
Confusion matrix obtained by the NFC.

The model was coded and implemented in the MATLAB environment (Matlab R2011b) and simulation results were then obtained. The NFC was trained with 100 iterations. In the study, a 10-fold cross-validation method was utilized to avoid overfitting. The training dataset was divided into 10 subsets. Each classifying structure was trained 10 times. Each time, one of the 10 subsets served as the validation set and the remaining subsets were used as the training sets. The classifying structure that was selected has the highest accuracy on the validation set (averaging over 10 runs). After training and validating, the NFC was tested using the testing dataset. Efficiency of the classifier was determined by comparing the predicted and actual class labels for the testing dataset. The comparison is given in Figure 3, in which the confusion matrix is represented.

The NFC was able to accurately predict 60 out of 71 for the “good”, 139 out of 148 for “average,” and 36 out of 42 for “poor.” This gives an accuracy of 84.51%, 93.2%, and 85.17% for “good,” “average,” and “poor” classifications, respectively. This provides 90.03% accuracy for the NFC, which is satisfactory when compared with results from studies on prediction.

**Figure 4:**
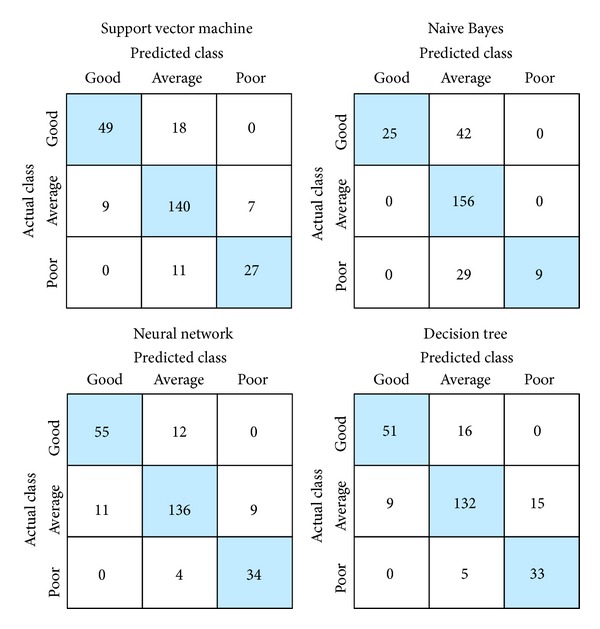
Confusion matrices obtained by different classification approaches.

To assess the performance of the NFC, we compared the results obtained by the NFC with those obtained by other classification approaches. The 10-fold cross-validation method was also used to identify the classifier structures. For the SVM, RBF kernel (often called Gaussian kernel) was used. The prediction accuracy of the SVM classifier for the testing dataset came out to be 82.76%. For the Naive Bayes classifier, the prediction accuracy of the classifier was found to be 72.8% for the testing dataset. In order to perform the classification based on a neural network, we investigated different neural network architectures with different numbers of hidden layers and neurons. Performance was measured using mean squared error function; the Levenberg-Marquardt algorithm was utilized to train neural networks. The network architecture with the highest efficiency in comparison with other architectures was selected. The architecture that was selected consisted of a single hidden layer with 10 neurons. Overall, accuracy of the neural network was 86.2%. Finally, a classifier based on a decision tree was applied to the problem. The classification and regression tree (CART) algorithm was used for constructing the decision tree model. The obtained accuracy for the decision tree was 82.76%. The results of these approaches to the classification of students' academic performance levels are summarized in Figure 4, together with confusion matrices. When these results were compared with those obtained by the NFC model, it was found that the NFC outperformed the SVM, Naive Bayes classifier, neural network, and decision tree in classifying students' academic performance levels.

From the results, it can be concluded that the NFC model can be used to classify students into different groups based on their expected academic performance levels. The model achieved an accuracy of over 90%, which shows that it may be acceptable and good enough to serve as a classifier of students' academic performance levels.

## 7. Conclusions

By classifying students into different groups, educational institutions are able to strengthen their admission systems as well as provide better educational services. Thus, a model which could classify students based on their expected academic performance levels is necessary for institutions. There have been various approaches to classifiers. However, for a specific problem, increasing the classification model accuracy is still a subject with great importance. In this study, we presented an NFC model to a group of students. We also evaluated the classification accuracy of the model by comparing it with other well-known classifiers, including SVM, Naive Bayes, neural network, and decision tree classifiers. The obtained results demonstrated that the NFC model outperformed the others. The results of the present study also reinforce the fact that a comparative analysis of different approaches is always supportive in choosing a classification model with high accuracy. It is expected that this study may be used as a reference for decision-making in the admission process and to provide better educational services by offering customized assistance according to students' predicted academic performance levels.

## Figures and Tables

**Table 1:** Input variables.

Input variable	Range
University entrance examination score	
Subject 1	0.5–10
Subject 2	0.5–10
Subject 3	0.5–10
The average overall score of high school graduation examination	5–10
Elapsed time between graduating from high school and obtaining university admission (0 year: 0; 1 year: 1; 2 years: 2; and 3 years and above: 3)	0, 1, 2, 3
Location of student's high school (Region 1: 0; Region 2: 1; Region 3: 2; and Region 4: 3)	0, 1, 2, 3
Type of high school attended (private: 0; public: 1)	0, 1
Student's gender (male: 0; female: 1)	0, 1

**Table 2:** Output class labels.

Class label	Class
1	Good
2	Average
3	Poor
